# Circulating tumor DNA analysis for prediction of prognosis and molecular insights in patients with resectable gastric cancer: results from a prospective study

**DOI:** 10.1002/mco2.70065

**Published:** 2025-01-19

**Authors:** Zheng Liu, Zhongyi Shi, Wenchao Jiang, Zhenbin Shen, Weidong Chen, Kuntang Shen, Yihong Sun, Zhaoqing Tang, Xuefei Wang

**Affiliations:** ^1^ Department of Gastrointestinal Surgery Zhongshan Hospital Fudan University Shanghai China; ^2^ Gastric Cancer Center Zhongshan Hospital Fudan University Shanghai China; ^3^ Shanghai Medical College Fudan University Shanghai China; ^4^ Department of General Surgery Zhongshan Hospital (Xiamen Branch) Fudan University Shanghai China

**Keywords:** circulating tumor DNA (ctDNA), gastric cancer (GC), prediction model, prognosis

## Abstract

This study aimed to evaluate the prognostic value of plasma circulating tumor DNA (ctDNA) level in patients with resectable gastric cancer (GC). A total of 59 patients were prospectively enrolled, with their ctDNA detected and paired tumor tissue collected at various peri‐operative time points. Patients with higher 1‐month post‐operative ctDNA levels demonstrated shorter overall survival status (hazard ratio [HR] = 5.30, *p* = 0.0022) and a higher risk of recurrence (HR = 3.85, *p* = 0.011). The model combining ctDNA with conventional serum tumor markers for GC, including carcinoembryonic antigen, carbohydrate antigen 19‐9, and CA72‐4, shows high predictive effectiveness for GC prognosis with an area under the curve of 0.940 (*p* = 0.002), which is higher than net ctDNA and other models without ctDNA. Patients with lower ctDNA levels were more likely to have positive stromal programmed cell death ligand 1 expression (*p* = 0.046). Additionally, DCAF4L2 mutation was identified as the crucial gene mutation in ctDNA suggesting poor prognosis of patients with GC. Overall, this study highlights that post‐operative ctDNA can serve as an effective biomarker for prognostic prediction and recurrence surveillance in resectable GC.

## Graphical abstract

Our study indicated ctDNA level in 1‐month post‐operative plasma sample effectively predicted prognosis of GC patients. Plasma samples and paired tumor tissue were prospectively collected from patients pre‐operatively at baseline and at subsequent time points of post‐operative follow‐up for ctDNA detection by AVENIO ctDNA Surveillance Kit. Integrated with clinicopathological information, prognostic prediction was conducted in further analysis.

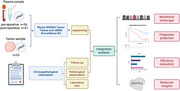

## 1 INTRODUCTION

Gastric cancer (GC) ranks as the fifth most common malignancy and the third leading cause of cancer‐related death worldwide.^1^ Although the 5‐year overall survival (OS) rate for early‐stage patients exceeds 60%, half of patients with GC are diagnosed in advanced stages, where the 5‐year OS rate decreases to approximately 30%.^2^, ^3^, ^4^ For patients with advanced GC, especially those at high risk of relapse, the key to improve their prognosis is to apply intensified treatment following early detection of recurrence.^5^ Currently, the primary factors for assessing recurrence risk were are predominantly based on pathological methods, including tumor‐node‐metastasis (TNM) staging system,^6^ molecular features investigation,^7^, ^8^, ^9^ and pathomics development.^10^, ^11^, ^12^ However, invasive pathological examinations are not suitable for real‐time monitoring of tumors in clinical practice.

Liquid biopsy has emerged as a non‐invasive and repeatable method to investigate tumor burden and genetic alterations in cancer patients by longitudinally monitoring changes at the cellular and molecular levels.^13^, ^14^, ^15^ Previous evidence has demonstrated that cell‐free DNA (cfDNA) or circulating tumor DNA (ctDNA) is a promising biomarker for guiding diagnosis and predicting prognosis.^16^ Notably, ctDNA positivity is associated with the disease stage, as ctDNA is rarely detected in early‐stage GC patients while frequently detected in advanced ones.^17^

Recently, occult micro‐metastasis or minimal residual disease (MRD) has been considered as a potential source of subsequent metastasis and relapse, contributing to the poor prognosis of advanced GC patients.^18^ The advent of ctDNA‐based assays has enabled the identification and characterization of MRD in individual patients with solid tumors, proven reliable in breast cancer, colon cancer, and lung cancer.^19^, ^20^, ^21^

However, research on the prognostic utility of ctDNA in GC is currently limited. A previous study reported the prognostic prediction using plasma ctDNA levels in GC patients receiving adjuvant chemotherapy^22^ and revealed that the ctDNA level detected after adjuvant chemotherapy, rather the baseline level, could effectively predict a high risk of recurrence in stage II/III GC patients. Another study focused on distinguishing ctDNA from other cfDNA alterations, which also suggested that ctDNA could serve as a predictive biomarker of the outcome of peri‐operative therapy in patient with GC.^23^

This study aimed to evaluate the predictive potential of ctDNA in the prognosis of patients with GC. Furthermore, the essential gene mutation in ctDNA closely associated with poor prognosis of GC was also identified for further in‐depth exploration.

## 2 RESULTS

### 2.1 Clinicopathological characteristics of patients enrolled with resectable GC

A total of 59 patients were enrolled in this study, with 168 longitude blood samples and 53 corresponding primary tumor samples. Fifty‐six patients had baseline blood samples collected pre‐operatively and 41 patients had blood samples collected in 1‐month post‐operatively during follow‐up (Figure S1A,B). Information on demographics and clinicopathological characteristics of patients are presented in Table 1. Most tumors were located in the stomach rather than the gastroesophageal junction. Approximately 40.68% of tumors had a maximum diameter of over 5 cm. Pathological examination revealed poorly differentiated tumors in 86.44% of cases, while the remaining cases were moderately to highly differentiated. Regarding the Lauren's classification, 15 patients (25.42%) had a diffuse type and 16 patients (27.12%) had intestinal type. Most patients were detected with lymphatic invasion (LVI) (71.19%). More than half (55.93%) of patients were diagnosed with an advanced stage (pathological tumor‐node‐metastasis [pTNM] III‐IV). Based on the The Cancer Genome Atlas Program (TCGA) molecular classification, two tumors were classified as Epstein–Barr virus (EBV) type (3.39%) and four tumors were classified as microsatellite instability (MSI) type (6.78%). The chromosomal instability (CIN) type accounted for 59.32% of total cases, while the remaining cases were classified as genomic stability (GS) type (30.51%). All 59 patients received surgery with curative intent, and 61.02% of them received adjuvant chemotherapy. As of the data cut‐off on February 18, 2022, the median follow‐up time was 746 (24–969) days. Twenty‐five (42.37%) patients had a recurrence, and 18 (30.51%) patients died from tumor‐related causes.

**TABLE 1:** Summary of baseline clinicopathologic characteristics.

Variable	Patients (*n* = 59)
Sex, *n* (%)	
Male	41 (69.49%)
Female	18 (30.51%)
Age, *n* (%)	
<60	12 (20.34%)
≥60	47 (79.66%)
Tumor location, *n* (%)	
Gastroesophageal junction	12 (20.34%)
Stomach	44 (74.58%)
Other	3 (5.08%)
Tumor size, *n* (%)	
<5 cm	35 (59.32%)
≥5 cm	24 (40.68%)
Lauren type, *n* (%)	
Diffuse	15 (25.42%)
Intestinal	16 (27.12%)
Mixed	22 (37.29%)
Undefined	6 (10.17%)
TCGA type, *n* (%)	
EBV	2 (3.39%)
MSI	4 (6.78%)
CIN	35 (59.32%)
GS	18 (30.51%)
Differentiation, *n* (%)	
Moderate to high	8 (13.56%)
Poor	51 (86.44%)
LVI, *n* (%)	
No	17 (28.81%)
Yes	42 (71.19%)
pTNM, *n* (%)	
I—II	25 (42.37%)
III	33 (55.93%)
NA	1 (1.69%)
Adjuvant chemotherapy, *n* (%)	
No	21 (35.59%)
Yes	36 (61.02%)
NA	2 (3.39%)

Abbreviations: CIN, chromosomal instability; EBV, Epstein–Barr virus; GS, genomically stability; LVI, lymphatic invasion; MSI, microsatellite instability; PNI, perineural invasion; pTNM, pathological tumor‐node‐metastasis; TCGA, The Cancer Genome Atlas Program.

### 2.2 The mutational landscape of genomic alterations in ctDNA and tumor tissue of resectable GC

**FIGURE 1:**
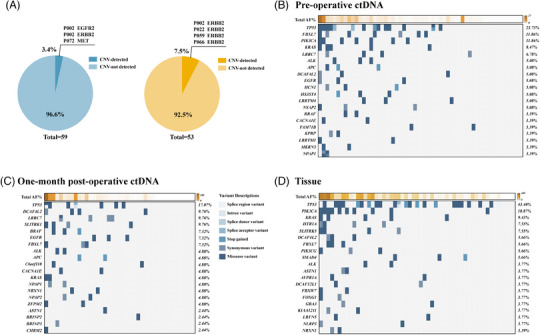
**Mutational landscape of genomic alterations in** circulating tumor DNA (ctDNA) **of detected in plasma samples and tumor tissue**. Pie charts illustrated the proportion of copy number variation in detected mutation by sequencing in ctDNA of plasma (left, *n* = 59, all phases included) and tumor tissue (right, *n* = 53) (**A**). P002 represented the patient was assigned the number two as the screening number in the study. Other codes could be interpreted similarly by analogy. The frequency of single nucleotide variants (SNVs) per gene were analyzed. Genes were then sorted and ranked by the frequency of SNV from the highest to the lowest. Top 20 genes, variant descriptions, and total AF value of each patient in ap00 (*n* = 56) (**B**), ap01 (*n* = 41) (**C**), and tumor tissue (*n* = 53) (**D**) were displayed in waterfall plots. AF, allele fraction. ap00: pre‐operative (baseline); ap01: 1‐month post‐operative.

To assess the concordance of genomic alterations between ctDNA in plasma samples from different phases and tumor tissue, genes were sequenced via the Roche AVENIO surveillance panel, a pan‐cancer panel that spans 198 kb and encompasses 197 genes. According to the captured NGS analysis, there was only a small subset of patients with detectable copy number variation in both blood samples (3.4%) and tumor tissue (7.5%) (Figure 1A). Notably, there was a distinct difference in Top20 genes ranked by mutation frequency between pre‐operative and post‐operative blood samples (Figure 1B–D). Nevertheless, the most frequently altered gene in tumor tissue as well as pre‐operative and post‐operative blood samples was *TP53*, with a frequency of 43.40%, 23.73%, and 17.07%, respectively. Missense variants were the predominant type of mutations observed in our cohort. The mutation frequencies of genes in our cohort were then further compared with the TCGA stomach adenocarcinoma (STAD) cohorts (Figure S2A). No obvious concordance was observed in genomic alterations between our cohort and TCGA cohort, which might be attributed to the limited number of detectable genes and different cohort backgrounds. The mutational frequency and average allele frequency (AF) both demonstrated a direct proportion total AF per patients, irrespective of pre‐operative and post‐operative blood samples or tumor tissue (Figure S2B–G).

### 2.3 One‐month post‐operative plasma ctDNA level effectively predicted prognosis

**FIGURE 2:**
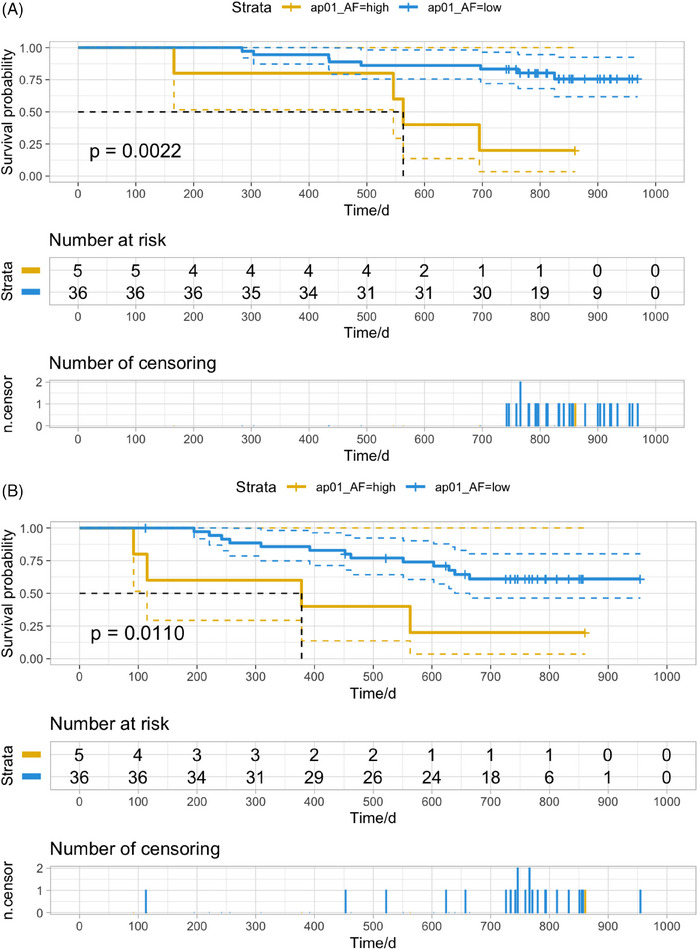
**Levels of** circulating tumor DNA (ctDNA) **allele fraction in post‐operative plasma samples were associated with prognosis of patients with resectable gastric cancer**. Kaplan–Meier curves of overall survival (OS) were drawn for patients with high level (*n* = 5) and low level (*n* = 36) of ctDNA allele fraction (AF) in ap01 plasma samples (**A**). The best cut‐off value of ap01 ctDNA AF for overall survival (OS) was 2.54%, determined by X‐tile software and R packages “survival” and “survminer”. The *p* value of log‐rank test was indicated. Kaplan–Meier curves of progression‐free survival (PFS) were drawn for patients with high level (*n* = 5) and low level (*n* = 36) of ctDNA AF in ap01 plasma samples (**B**). The best cut‐off value of ap01 ctDNA AF for PFS was 2.54%. The *p* value of log‐rank test was indicated. ap00: pre‐operative (baseline); ap01: 1‐month post‐operative.

Correlation analysis and survival analysis were then conducted to investigate the potential of ctDNA as a promising biomarker in the prediction of prognosis. No significant differences in clinicopathological characteristics were observed between patients with positive versus negative pre‐operative or post‐operative ctDNA results (Table 2). Multivariable analysis identified 1‐month post‐operative ctDNA as an independent factor for both OS and progression‐free survival (PFS) (Table 3). Among 41 patients with plasma samples collected and underwent ctDNA detection within 1 month after surgery, five (12.2%) patients had high‐level ctDNA, while 36 (87.8%) had low‐level ctDNA. A significant reduction of OS was observed in patients with high‐level ctDNA compared to those with low‐level ctDNA (Figure 2A, hazards ratio [HR] = 5.30, *p* = 0.0022). Patients with high‐level ctDNA tended to have a higher risk of recurrence (Figure 2B, HR = 3.85, *p* = 0.0110). The level of ctDNA AF in patients who died during follow‐up was numerically higher than in those who survived (Figure S3A). Similarly, patients who experienced disease progression during follow‐up were more likely to have a higher level of ctDNA AF than those who did not (Figure S3D). There was a slight decrease in ctDNA level in 1‐month post‐operative plasma samples compared to pre‐operative samples from patients with good prognosis (Figure S3B,E). An elevation of ctDNA level was observed in some patients who died or experienced relapse during follow‐up (Figure S3C,F). These findings indicated the dynamic change of ctDNA level was influenced to a certain extent by therapeutic interventions such as surgery. Moreover, the periodical monitoring of ctDNA in plasma samples could effectively reflect treatment response and predict prognosis effectively.

**TABLE 2:** Clinicopathologic characteristics as per circulating tumor DNA (ctDNA) positivity.

Variable	Pre‐operative ctDNA (ap00)			Post‐operative ctDNA (ap01)		
	Negative (*n* = 11)	Positive (*n* = 45)	*p*‐value	Negative (*n* = 11)	Positive (*n* = 30)	*p*‐value
Sex			0.719			0.463
Male	7	32		6	21	
Female	4	13		5	9	
Age			>0.999			>0.999
<60	2	10		1	4	
≥60	9	35		10	26	
Tumor location			0.434			0.083
AEG	1	10		0	8	
Stomach	10	35		11	22	
Tumor size			>0.999			>0.999
<5 cm	7	27		6	17	
≥5 cm	4	18		5	13	
Lauren type			0.773			0.363
Diffuse	4	10		4	5	
Intestinal	2	13		4	8	
Mixed	4	18		2	13	
Undefined	1	4		1	4	
TCGA type			0.816			0.283
EBV	0	2		0	1	
MSI	1	3		1	1	
CIN	6	28		5	22	
GS	4	12		5	6	
Differentiation			>0.999			0.316
Moderate to high	1	6		3	3	
Poor	10	39		8	27	
LVI			>0.999			0.719
No	3	13		4	9	
Yes	8	32		7	21	
pTNM			0.067			0.166
I and II	6	18		6	13	
III	4	27		4	17	
NA	1	0		1	0	
Adjuvant chemotherapy			0.559			0.588
No	5	14		3	12	
Yes	6	29		8	17	
NA	0	2		0	1	

**Abbreviations**: AEG, adenocarcinoma of esophago‐gastric junction; ap00: pre‐operative (baseline); ap01: 1‐month post‐operative; CIN, chromosomal instability; EBV, Epstein–Barr virus; GS, genomically stability; LVI, lymphatic invasion; MSI, microsatellite instability; PNI, perineural invasion; pTNM, pathological tumor‐node‐metastasis; TCGA, The Cancer Genome Atlas Program.

**TABLE 3:** Univariate and multivariate analyses of the endpoints and associated clinicopathologic characteristics.

OS	Univariate model			Multivariate model		
Variable	HR	95% CI	*p*‐value	HR	95% CI	*p*‐value
Sex						
Female	1 (ref)					
Male	0.84	(0.21, 3.37)	0.805			
Age	0.96	(0.89, 1.03)	0.282			
AEG						
No	1 (ref)					
Yes	0.61	(0.13, 2.94)	0.536			
Tumor size	1.43	(1.15, 1.78)	**0.001** ^*^	2.80	(2.10, 3.90)	**0.000** ^*^
Lauren type						
Diffuse	1 (ref)					
Non‐diffuse	0.16	(0.04, 0.58)	**0.006** ^*^			
LVI						
No	1 (ref)			1 (ref)		
Yes	3.52	(0.44, 28.19)	0.237	110	(9.80, 1300)	**0.000** ^*^
pTNM						
I and II				1 (ref)		
III	1.52E+09	(0, Inf)	0.999	3.10E+08	(0, Inf)	1.000
TCGA type						
GS	1 (ref)					
Non‐GS	0.28	(0.07, 1.03)	0.055			
Adjuvant chemotherapy						
No	1 (ref)					
Yes	4.65	(0.57, 38.19)	0.152			
ap00_AF%	1.43	(0.83, 2.46)	0.196	0.57	(0.15, 2.20)	0.410
ap01_AF%	1.32	(1.03, 1.69)	**0.031** ^*^	2.00	(1.50, 2.80)	**0.049** ^*^

PFS	Univariate model			Multivariate model		
Variable	HR	95% CI	*p*‐value	HR	95% CI	*p*‐value
Sex						
Female	1 (ref)					
Male	0.73	(0.21, 2.50)	0.617			
Age	0.94	(0.88, 1.01)	0.105			
AEG						
No	1 (ref)					
Yes	0.90	(0.24, 3.38)	0.872			
Tumor size	1.27	(1.07, 1.51)	**0.006** ^*^	1.40	(1.10, 1.70)	**0.011** ^*^
Lauren type						
Diffuse	1 (ref)					
Non‐diffuse	0.23	(0.07, 0.80)	**0.020** ^*^			
LVI						
No	1 (ref)			1 (ref)		
Yes	1.82	(0.39, 8.41)	0.446	0.89	(0.10, 7.60)	0.910
pTNM						
I‐II	1 (ref)			1 (ref)		
III	8.35	(1.07, 65.52)	**0.043** ^*^	5.20	(0.40, 68)	0.210
TCGA type						
GS	1 (ref)					
Non‐GS	0.26	(0.08, 0.86)	**0.027** ^*^			
Adjuvant chemotherapy						
No	1 (ref)					
Yes	2.60	(0.55, 12.24)	0.228			
ap00_AF%	1.32	(0.96, 1.81)	0.090	0.10	(0.01, 0.93)	**0.043** ^*^
ap01_AF%	1.46	(1.08, 1.97)	**0.014** ^*^	4.00	(1.30, 12)	**0.014** ^*^

* The bold values indicate *p*‐value < 0.05.

### 2.4 ctDNA combining with tumor markers achieved a good efficacy in prognostic prediction

**FIGURE 3:**
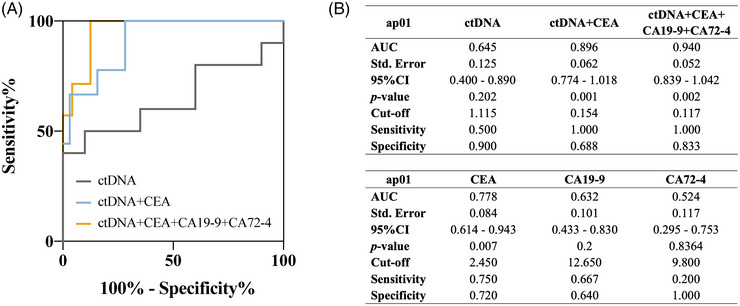
**One‐month post‐operative plasma** circulating tumor DNA (ctDNA) **level as a prospective factor to predict of 3‐year survival status**. Receiver operating characteristics (ROC) analysis of net ctDNA (black), the combination of ctDNA and CEA (blue), and the combination of ctDNA and the three conventional tumor markers of gastric cancer, including CEA, CA 19‐9, CA 72–4 (red) for predicting 3‐year survival status (**A**). A summary of the predictive efficacy obtained in ROC analyses (**B**). ROC, receiver operating characteristics; AUC, area under curve; CEA, carcinoma embryonic antigen; CA, carbohydrate antigen; Std. Error, standard error; CI, confidence interval.

To evaluate the efficacy of prognostic prediction of ctDNA level, the receiver operating characteristics (ROC) analysis was conducted to predict the outcome of prognosis such as survival or recurrence. When conducting ROC for prognostic prediction of post‐operative ctDNA, the “survival” was not a time‐dependent factor but a binary factor revealing the survival status: living or dead. In terms of the predictive efficacy for survival reported by ROC, ctDNA from the plasma sample yielded an area under the curve (AUC) of 0.645. At a cut‐off value of 1.115%, ctDNA achieved a sensitivity of 50.0% and a specificity of 90.0% (Figure 3A,B). When combining ctDNA and conventional serum tumor markers such as carcinoembryonic antigen (CEA), CA (carbohydrate antigen) 19‐9, and CA 72‐4, a significantly higher efficacy than ctDNA or any tumor markers alone was observed. Particularly, the combination of ctDNA and tumor markers that patients with GC were often recommended to detect in regular examination during follow‐up (CEA, CA19‐9, and CA72‐4) presented a remarkably higher efficacy with an AUC of 0.940 (*p* = 0.002). Regarding the predictive efficacy for 3‐year‐survival status reported by ROC, plasma ctDNA level yielded an AUC of 0.645. A slight increase in AUC after adding pTNM stage or tumor size to the model combining ap01 ctDNA level and tumor markers can be observed in Table S1, indicating that the predictive effect for survival could be further enhanced by incorporating conventional clinicopathological information. Regarding recurrence prediction, net ctDNA demonstrated an unsatisfactory performance with an AUC of 0.591, comparable to three biomarkers alone (Figure S4A). However, the combination of ctDNA and tumor markers achieved an AUC = 0.852 (*p* = 0.010) (Figure S4C). Fitting analysis revealed that ctDNA levels were nearly independent of serum tumor markers like CEA and CA19‐9, whereas it positively correlated to CA72‐4 in 1‐month post‐operative plasma samples (Figure S4G–I). This suggested ctDNA could serve as an independent predictive factor exhibiting greater potential in prognostic prediction, especially when combining with clinicopathological and laboratory information.

### 2.5 Post‐operative ctDNA level moderately reflected stromal PD‐L1 and PD‐1 expression status

**FIGURE 4:**
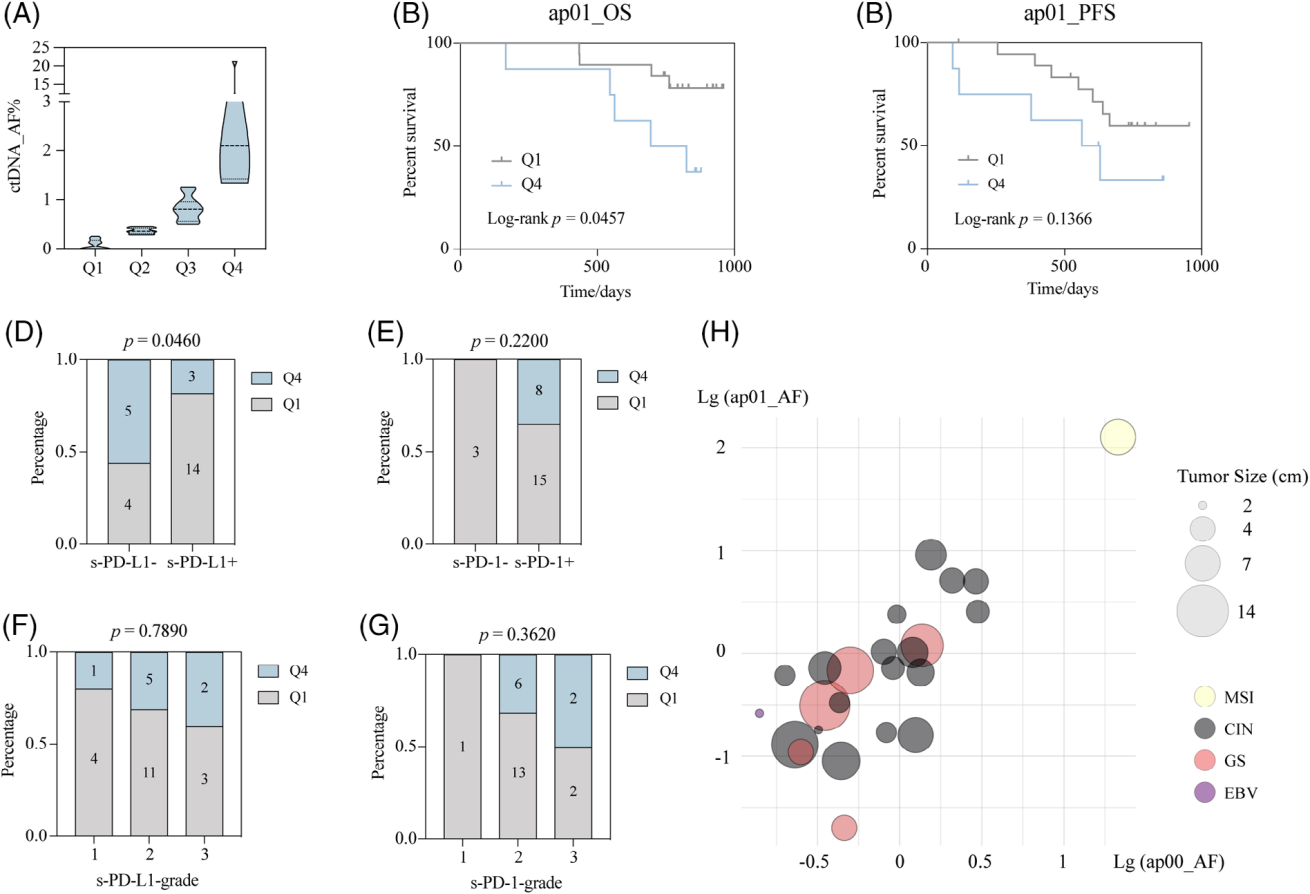
**The associations between levels of** circulating tumor DNA (ctDNA) **AF in post‐operative plasma samples and molecular features in patients with gastric cancer**. Patients were grouped in four quantiles Q1–Q4, depending on the mean level of ctDNA allele fraction (AF) (**A**). Kaplan–Meier curves of overall survival (OS) (**B**) and progression‐free survival (PFS) (**C**) were drawn for patients with ctDNA AF level in quantiles Q1 (*n* = 10) versus Q4 (*n* = 12). The *p* value of log‐rank test was indicated. A significant correlation between levels of ap01 ctDNA AF and programmed death‐ligand 1 (PD‐L1) status in stroma could be observed (**D**), while there was no significant correlation between levels of ap01 ctDNA AF and programmed death‐1 (PD‐1) status in stroma (**E**). Patients with a higher stromal PD‐L1 grade and PD‐1 grade were more likely to attain a higher elevation in post‐operative period (**F** and **G**). The number labeled on the bar represented the number of samples included. The bubble plot showed the relation between tumor size, The Cancer Genome Atlas (TCGA) molecular subtypes, and ctDNA AF level in ap00 and ap01 plasma sample (**H**). The size of bubble represented tumor size. Different colors indicated four TCGA subtypes. t‐PD‐L1, tumor‐PD‐L1; s‐PD‐L1, stromal‐PD‐L1.

The molecular features of tumor and their microenvironment were predominant factors in determining therapeutic response and disease progression, which often function as indispensable references for clinical decision making. Patients were divided into four groups according to quartile of ctDNA level (Figure 4A). Patients in Q4 had a higher risk of progression and were more likely to have a shorter OS compared to those in Q1 (Figure 4B,C). It was illustrated by subgroup analysis that patients who had positive stromal programmed death‐ligand 1 (PD‐L1) expression in pathological examination tended to have lower ctDNA level post‐operatively (*p* = 0.046) (Figure 4D). Nevertheless, patients with positive stromal programmed death‐1 (PD‐1) expression appeared to have higher post‐operative ctDNA level (Figure 4E). Among patients with positive stromal PD‐L1 or PD‐1 expression, the grade showed a numerical but insignificant direct proportion with ctDNA level (Figure 4F,G). These results indicates that ctDNA could potentially provide some medication guidance for the long‐term application of immunotherapy via dynamic monitoring. However, due to the limited sample size, these findings had limited statistical meaning and needed further validation in future investigations. In addition, TCGA molecular classification was included in analysis (Figure 4H). Most patients had CIN‐type GC. Patients with GS‐type were more likely to have larger tumor sizes, represented by the longest diameter, while patients with EBV‐positive type exhibited the opposite trend. One patient with extremely high ctDNA level in pre‐operative and post‐operative plasma samples had MSI‐type GC.

### 2.6 *DCAF4L2* mutation status in ctDNA and correlated tumor tissue with poor prognosis

**FIGURE 5:**
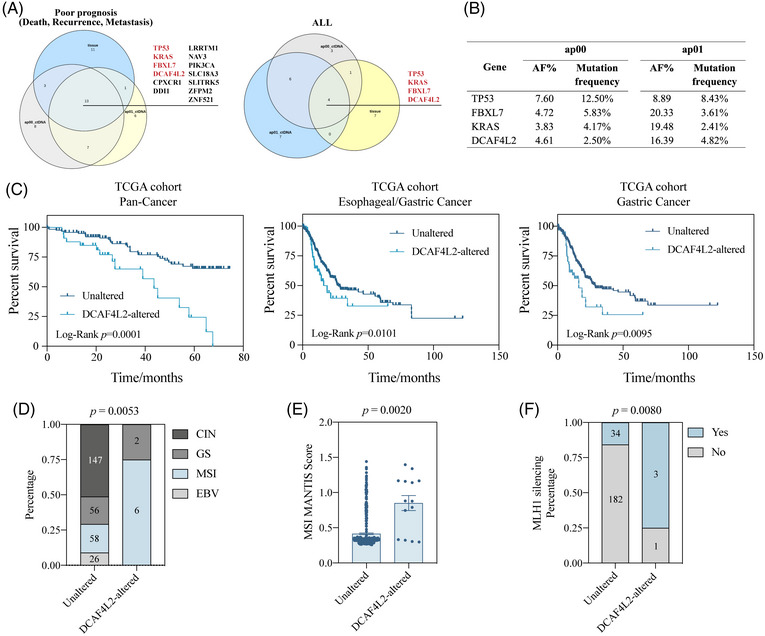
***DCAF4L2* was identified as a crucial gene for poor prognosis in patients with gastric cancer**. The candidate genes were presented by Venn diagram (**A**). Thirteen genes were overlapped in mutations detected in ap00 circulating tumor DNA (ctDNA), ap01 ctDNA, and tumor tissue in patients with poor prognosis (left). Furthermore, four genes were overlapped in all patients, all of which were included in poor prognosis‐related mutated genes mentioned above (right). The table showed the mutation frequency of four candidate genes in ap00 ctDNA and ap01 ctDNA (**B**). Kaplan–Meier curves of overall survival were drawn for patients with unaltered group and *DCAF4L2*‐altered group in Pan‐cancer (**C** left), esophageal/gastric cancer (**C** middle), and only gastric cancer cohort (**C** right) from TCGA database. The *p* value of log‐rank test was indicated. Patients in *DCAF4L2*‐altered group accounted for a significantly larger proportion of MSI‐type gastric cancer than unaltered group in TCGA cohort (**D**). The MSI MANTIS scores were significantly higher in *DCAF4L2*‐altered group (*n* = 14) than unaltered group (*n* = 421) in TCGA cohort (**E**). A large proportion of patients in *DCAF4L2*‐altered group had MLH1 silencing in TCGA cohort (**F**). The number labeled on the bar represented the number of samples included. MSI, microsatellite instability.

In addition, the specific gene mutation in ctDNA that had strong connection with poor prognosis were identified. It may offer us a novel approach to monitor disease progression by tracking the abundance of certain mutations in the plasma sample. The mutational status of ctDNA and tumor tissue in all enrolled patients as well as in patients with poor prognosis were screened, respectively (Figure 5A). The mutation of 13 genes were detected in both tumor tissue and ctDNA from pre‐ and post‐operative plasma samples in patients with poor prognosis. Among 13 genes, the mutation of four genes (*TP53*, *KRAS*, *FBXL7* and *DCAF4L2*) were shared by ctDNA and tumor tissue of all patients. The AF value and mutation frequency of these four genes from different sample along with mutation status in TCGA STAD cohort were displayed (Figure 5B and Figure S5A). The external cohort from TCGA database was utilized to explore the potential gene mutation related to poor prognosis. *DCAF4L2* was selected as the candidate gene due to its mutation being associated with shorter OS in the pan‐cancer cohort, esophageal/GC cohort, and only GC cohort (Figure 5C). In our cohort, post‐operative plasma samples from patients with *DCAF4L2* alterations showed slightly higher ctDNA levels compared to those without alterations (Figure S5B). Subgroup analysis was then applied in TCGA STAD cohort to discover the mechanism behind. Patients with *DCAF4L2* alteration in tumor tissue occupied a larger proportion of MSI‐type GC with a significantly higher average MSI MANTIS Score compared to those unaltered ones, which was in consistent with the pattern in our cohort (Figure 5D,E and Figure S5C). Unfortunately, restricted to a small sample size, our results failed to exhibit the significance due to only five DCAF4L2‐altered patients in our cohort. MLH1 silencing, a well‐acknowledged essential event in MSI GC that was mainly caused by methylated modification, was found to occur more frequently in patients with DCAF4L2 mutations (Figure 5F). To confirm the methylation status of MLH1 in MSI‐type GC in the TCGA STAD cohort, CpG island methylator phenotype data were derived and analyzed (Figure S5D). The methylation level of MHL1 was higher in *DCAF4L2*‐altered patients than unaltered ones in several detections via different probes (Figure S5E–I). The Kyoto Encyclopedia of Genes and Genomes (KEGG) analysis showed that multiple common pathways in GC, such as neuroactive ligand–receptor interaction, WNT signaling pathway, proteoglycans in cancer, and cell adhesion molecules, were top enriched, which provides biological explanations on the mechanism of *DCAF4L2* mutation exacerbating the disease (Figure S5J). Although the DCAF4L2 mutation can identify patients with poor prognosis, its contribution to the predictive efficacy is minimal (Table S1). This implied that the prognostic prediction could be achieved more accurately and promptly through detecting specific gene mutations in ctDNA from plasma samples.

## 3 DISCUSSION

This study suggests that that the 1‐month post‐operative plasma ctDNA level can effectively predicted the prognosis of patients with resectable GC. The molecular landscapes of plasma ctDNA and the paired tumor tissue were characterized through detecting gene mutation using a 197‐gene panel. Multivariate analysis indicated that a high plasma post‐operative ctDNA level is strongly correlated to poor prognosis and thus can serve as an independent factor, suggesting that patients with high post‐operative ctDNA level tended to have a shorter OS and PFS. Although post‐operative ctDNA alone was not sufficient for accurate prognosis prediction, the combination of ctDNA and conventional serum tumor markers for GC, including CEA, CA19‐9 and CA72‐4, determined to efficiently predict OS with a high AUC of 0.940. Further investigation was conducted to explore the relationship between ctDNA and molecular features of the tumor. Patients with elevated ctDNA levels exhibited a lower likelihood of positive stromal PD‐L1 expression, whereas an opposite trend was noted for stromal PD‐1 expression. The preliminary association between TCGA molecular classification and post‐operative ctDNA level was determined. Furthermore, *DCAF4L2* might be a potential gene whose mutation is critical for diagnostic purpose. *DCAF4L2* mutation was closely relevant to MSI‐type GC, as patients with *DCAF4L2* alteration exhibited higher MSI MANTIS scores and a greater likehood of MLH1 silencing, typically caused by elevated MLH1 methylation level. This provides a novel approach, enabling the dynamic monitoring of disease progression and prognosis prediction in GC via precise detection of specific gene mutations in ctDNA.

The promising predictive value of ctDNA in the prognosis of malignant tumor has been sufficiently demonstrated in digestive tract cancers. One recent study showed that a significant difference in 3‐year recurrence‐free survival (RFS) rate was observed in patients with detectable versus undetectable ctDNA after surgery (47% vs. 76%) and after adjuvant chemotherapy (30% vs. 77%) in stage III colon cancer.^24^ Another study in esophageal cancer illustrated that the positive ctDNA result detected after radiochemotherapy was associated with tumor progression, the formation of distant metastases, and reduced disease‐specific survival time.^25^ Regarding the application of ctDNA in GC, the latest study emphasized that patients with positive post‐operative ctDNA results were at higher risk of recurrence compared to ctDNA‐negative patients. Furthermore, no significant difference in RFS or OS difference was observed between patients with and without detectable pre‐operative ctDNA.^22^

However, the definition of positive ctDNA result is varied in literature and clinical pratice. In several published studies on applying ctDNA in gastrointestinal tumors, each study employed a distinct approach to defining ctDNA positivity.^22^, ^23^, ^26^, ^27^, ^28^, ^29^ Each method had its rationale, making the definition of ctDNA positivity a prominent challenge in this field. This leads to variability in the definition of ctDNA positivity across different studies and results in different perspectives on the predictive capabilities for ctDNA in prognosis. For patients with a positive ctDNA result, it remains unsettled whether the quantity or this positive result is sufficient for prognostic prediction. A prior investigation suggested that higher cut‐offs for ctDNA mutation/mL of plasma samples were not strongly associated with higher risk of relapse, indicating that the presence of ctDNA rather than its load is more related to higher recurrence risk.^22^ Furthermore, some patients with a negative ctDNA detection result throughout the follow‐up period eventually developed disease progression, resulting in shorter survival times. This could likely be attributed to MRD, which occasionally falls below the minimum detection threshold of current ctDNA methods. Currently, MRD detection and characterization mainly rely on circulating tumor cells and ctDNA detection, which are limited by available resources and enrichment methods to a certain degree.^18^ The continuous refinement and expansion of liquid biopsy methods will contribute to the dynamic monitoring of ctDNA with a higher sensitivity.

PD‐L1 can be expressed by tumor cells and tumor stroma, while PD‐1 is one of the co‐inhibitory receptors predominantly expressed on the surface of antigen‐stimulated T cells within the tumor stroma.^30^ Additionally, high expression of PD‐1 is one of the defining characteristics of exhausted T cells.^31^ In exhausted T cells, PD‐1 maintains the dysfunctional state within the tumor.^32^ Our analysis revealed that patients with elevated ctDNA levels exhibited a lower likelihood of positive stromal PD‐L1 expression, whereas the trend for stromal PD‐1 expression is the opposite. This observation suggests that patients with higher ctDNA levels may possess immunosuppressive tumor microenvironments, likely related to T‐cell dysfunction.^33^ According to a previous study, patients with immunosuppressive tumors were more likely to experience recurrence.^34^, ^35^ These studies suggest that a higher post‐operative ctDNA level can indicate a higher risk of recurrence due to the immunosuppressive TIME revealed by high expression of PD‐1 in tumor stroma. Overall, post‐operative plasma ctDNA levels could serve as a biomarker for guiding the utilization of PD‐1 inhibitors in clinical practice, particularly given their association with stromal PD‐1 expression to a certain degree.

This study revealed that the combination of ctDNA and other indexes significantly increased the prediction ability of prognosis compared with ctDNA alone, which was consistent with several previous studies. A previous study concluded that the combination of ctDNA and T stage could effectively discriminate patients with a higher risk of recurrence from all enrolled patients with colon cancer.^29^ Another prospective study in patients with locally advanced rectal cancer established a risk score predictive model that incorporated both ctDNA and magnetic resonance imaging (MRI) tumor regression grade, which improved the performance with an AUC of 0.886 compared to the models derived from individual information. Additionally, plenty of pathological features, including perineural invasion, tumor deposit, and vascular invasion, were defined as a “high‐risk feature (HR feature)” status. The combination of HR features and ctDNA could stratify patients with a high risk of recurrence.^36^ These results suggested that a single indicator could not describe every perspective reflecting how the prognosis of the tumor was impacted. The integration of multilevel information, including clinicopathological characteristics and laboratory inspection results, offered an optimal combination to optimize the current predictive model of prognosis.

One possible reason for the dissatisfactory efficacy of ctDNA alone in the prediction of prognosis could be ascribed to the inaccurate detection of gene mutation in ctDNA. This study screened a specific gene mutation in post‐operative ctDNA closely related to poor survival outcome, which reminded us that a more credible judgment of disease progression could be made via detecting mutation of a few crucial genes. Plenty of research utilized commercial kits with a panel containing at least hundreds of genes for a comprehensive ctDNA detection, which inevitably led to a rise of false positive rate and detection rate of “background” gene mutation, which has weak associations with the objective of prognostic prediction. The “background” gene mutation was a relative concept, which means the gene mutation has limited significance for the purpose of detection. This notion aligns with some studies on the clinical application of ctDNA. Zhu et al. selected the median variant allele fraction level of *NRAS*, *NEF2L2*, and *MET* mutations in ctDNA as a moderately effective factor to predict early recurrence of hepatocellular carcinoma with AUC of 0.80, while the AUC achieved 0.97 when further combined with clinical parameters, including barcelona clinic liver cancer (BCLC) stage, tumor size, and microvascular invasion (MVI).^37^ This suggests that the concept of accurate, non‐invasive, and real‐time monitoring of MRD for the evaluation of therapeutic effects and disease progression is becoming acceptable and prevailing.

Moreover, DCAF4L2 was identified as a potential gene whose mutation was essential for prognostic prediction for resectable GC in this study. Correlation analysis was conducted to investigate the association between DCAF4L2 mutation and MSI status of GC. The full name of DCAF4L2 is DDB1 and Cul4 associated factor 4 like 2, which belongs to WD domain repeat containing protein family and functions as a subunit responsible for substrate recognition in the E3 ligase complex.^38^ DCAF4L2 was reported to participate in the regulation of histone methylation and protein expression.^39^ One previous study also suggests that DCAF4L2 is involved in DNA damage and repair processes, which are related to MSI status to a certain extent.^40^ However, the relation between MSI status and DCAF4L2 mutation has not been established yet. To investigate further, we searched cBioPortal (http://www.cbioportal.org) to find the mutation hotspot of DCAF4L2 in three GC cohorts (TCGA, GDC; TCGA, Firehose Legacy; TCGA, Nature 2014). A316T and L159P are the most frequently detected mutation hotspot (protein change) with high AF and both of them are missense mutations. However, no previous studies have reported pathogenic mutation sites of DCAF4L2, nor provided detailed information of theses mutation hotspot. There remain data gaps that require future investigations to address.

This study had some limitations, mainly attributed to the small sample size and moderately short follow‐up time. Two main findings in our study: the predictive value of ctDNA combined with conventional serum tumor markers for recurrence and the identification of DCAF4L2 mutation as the crucial gene in ctDNA for poor prognosis of resectable GC require further internal and external validations for their extrapolation in clinical decision‐making. Considering that the AVENIO Tumor Tissue and ctDNA surveillance kit demonstrated good analytical performance in previous studies, this study was not designed to include a control group. However, we encourage researchers to include control subjects in future ctDNA studies to ensure the stability of the analytical performance of the ctDNA kit in the study population and to provide a more comprehensive understanding of the findings. Moreover, pan‐cancer ctDNA surveillance kit was used rather than GC‐specific gene panel, which might fail to detect GC‐specific driver gene mutations. Limited by the scale of the study and insufficient evidence, the definition of ctDNA positivity in our study was inevitably inaccurate and not validated in a large cohort.

In conclusion, 1‐month post‐operative plasma ctDNA level was an independent factor of poor prognosis in patients with GC, including shortened OS and high risk of recurrence. The combination of post‐operative ctDNA level and conventional serum tumor markers in GC, including CEA, CA19‐9 and CA125, demonstrated good effectiveness in prognostic prediction. Additionally, the *DCAF4L2* mutation was identified as a crucial gene mutation in ctDNA, strongly correlated with poor prognosis, while the detailed underlying mechanism still requires further in‐depth investigation. Overall, these results affirm ctDNA as a promising biomarker for dynamic monitoring and surveillance of disease progression in resectable GC.

## 4 METHODS

### 4.1 Study design and patient cohort

This prospective study enrolled patients with GC from June 2019 to January 2020 in the GC Center of Zhongshan Hospital, Fudan University (Figure S1A). Patients were diagnosed according to standardized diagnostic criteria and clinical guidelines.^5^ Radical resection for GC was performed for the included patients. Patients were scheduled for periodical staging computed tomography of chest, abdomen, and pelvis during the follow‐up to exclude metastatic disease and relapse. Plasma samples and paired tumor tissue were also collected according to the prescribed procedure presented in the diagram (Figure S1B). This study is strictly in accordance with the Declaration of Helsinki and was approved by the Research Ethics Committees of Zhongshan Hospital (No. B2018‐264). Written informed consent was obtained from each patient.

### 4.2 Eligibility criteria

The inclusion criteria were as follows: (1) patients with pathologically confirmed adenocarcinoma in stomach or gastroesophageal junction and (2) age above 20 years.

The exclusion criteria were as follows: (1) cancer of other origins than stomach, such as melanoma, (2) patients who had received pre‐operative chemotherapy, (3) patients with radiological diagnosis of distal metastasis before surgery, or (4) patients with unresectable GC.

### 4.3 Sample collection and ctDNA extraction

Blood samples for ctDNA analysis and tumor marker detection were collected pre‐operatively (baseline) and post‐operatively during follow‐up, including the first month after surgery and every 3 months after that. At each time point, 10 mL of blood was drawn into a Roche Cell‐Free DNA Collection tube (Roche Diagnostics), and plasma was separated from the cells by centrifugation (1600 × *g* for 10 min at 4°C) followed by a second centrifugation of the supernatant at 16,000 × *g* for 10 min at 4°C to remove all cell debris. Plasma samples were frozen at −80°C until further processing in one batch.

### 4.4 Tumor tissue mutational analysis and ctDNA analysis

The AVENIO Tumor Tissue and ctDNA Surveillance Kit (Roche) covering 197 cancer‐related genes were used for analyses. Formalin‐fixed, paraffin‐embedded (FFPE) tumor tissue from the surgical specimen was analyzed for somatic mutations by next‐generation sequencing (NGS). Tumor sequencing was performed with 10–50 ng of FFPE DNA. The cfDNA from plasma was isolated using the Roche AVENIO ctDNA Surveillance Kit (Roche) according to the manufacturer's protocol. Sequencing was performed using 10–50 ng of cfDNA. Median de‐duped sequencing depths for cfDNA and FFPE DNA were 5360X and 2099X, respectively. Variants were identified by the AVENIO ctDNA Analysis Software that incorporates bioinformatics methods from CAPP‐Seq2 and iDES3 (integrated digital error suppression) to remove PCR duplicates and stereotypical errors from technical artifacts. Somatic variants were derived after filtering out common germline variants in public databases such as ExAC, dbSNP, and 1000 Genomes. AF for a given somatic mutation was calculated as the number of deduplicated reads with that mutation divided by the total number of deduplicated reads covering that genomic position. The validity of the lower limit of detectable AF of 0.2%. Only nonsynonymous single nucleotide variants (SNVs) or small insertions/deletions with an AF of at least 0.2% and 5% were included in further analyses for cfDNA and FFPE, respectively.

For further ctDNA quality control analysis, AF percentage was defined as the representative index of ctDNA level. The ctDNA results with an AF ≥ 40% were excluded for the abatement of clonal hematopoiesis with indeterminate potential. The mutation below the detection threshold presented in the detection report was considered as ctDNA negative, while the positive result was determined as ctDNA positive. Patients were divided into subgroups of high or low level of ctDNA. The best cut‐off values of ctDNA at different phase for OS and PFS were calculated by X‐tile software and R packages “survival” and “survminer”. In detail, the best cut‐off values of pre‐operative ctDNA were 1.38% and 1.34% for OS and PFS, respectively. The best cut‐off values of post‐operative ctDNA for OS and PFS were both 2.54%.

### 4.5 Histopathological assessment

Pathological characteristics were collected, including tumor location, size, differentiation, Lauren type, and LVI. The molecular classification of GC in this study is based on TCGA standard.^41^ Specifically, EBV status was examined by EBV‐encoded RNA in situ hybridization. Representative proteins in mismatch repair system, such as MLH1, MSH2, MSH6, and PMS2, were detected by immunohistochemistry (IHC) for determination of MSI status. Any of the proteins detected negative would be determined as mismatch repair deficiency (dMMR), while all proteins positive would be determined as mismatch repair proficiency. The dMMR was classified as the MSI‐type GC according to TCGA classification. GS GCs were defined as MSI or EBV‐negative diffuse‐type GCs. CIN GCs were identified if any of the classification standards above could not be met.

The expression level of PD‐1 and PD‐L1 in tumor and stroma tissue was determined via IHC assays (antibody: PD‐L1 clone 22C3, M3653, DAKO; PD‐1, ab137132, Abcam) by experienced pathologists.

### 4.6 Analysis based on the TCGA database

The primary data of genomic alterations in the TCGA STAD cohort (431 samples) was derived from the TCGA database (https://portal.gdc.cancer.gov) with the information of single nucleotide polymorphism. The visualization of mutations in the TCGA STAD cohort was conducted using the “*maftools*” package of R. The information on overall survival in different cohorts and relative expression levels of specific genes in both adjacent tissue and tumor were collected from the TCGA database. The data OncoPrint, clinicopathological characteristics, and methylation ChIP‐Seq (Chromatin Immunoprecipitation sequencing) of TCGA STAD were obtained from cBioPortal for Cancer Genomics (https://www.cbioportal.org) to validate the relevance between DCAF4L2 mutation and MSI status. The survival data of the external cohort were also derived from cBioPortal: Pan‐cancer analysis of whole genomes (ICGC/TCGA, Nature 2020), esophageal and gastric carcinoma (Nature 2017; PanCancer Atlas; Firehose Legacy; Nature 2014), and gastric carcinoma (PanCancer Atlas; Firehose Legacy; Nature 2014). The bioinformatics analysis of the KEGG pathway was conducted using the packages of R. The gene alteration status in TCGA dataset was defined as a general concept including amplification, deletion, and gain or loss of function.

### 4.7 Statistical analysis

The prognosis outcomes of the study were the OS and PFS of patients with different pre‐operative and post‐operative plasma ctDNA levels. The distribution of clinicopathological characteristics was compared between two groups by the Wilcoxon rank‐sum test. Pearson's chi‐square test was used to test the associations between the presence of ctDNA and clinicopathological information. Student's *t*‐test was used to assess the difference of ctDNA level between subgroups of plasma samples. Survival analyses were conducted using the Kaplan–Meier method. The cut‐off values were calculated, and survival plots with *p*‐value were calculated using the packages of R. Univariate Cox regression analysis was carried out to explore which parameters were associated with OS and PFS. Variables and parameters with *p*‐value < 0.02 were generally considered to indicate a poor prognosis, such as pTNM and LVI. These could further enter multivariate analysis. Spearman correlation coefficients were determined for the association between ctDNA AF and tumor markers. Visualizations of TCGA gastric adenocarcinoma cohort mutations were carried out in R. In this study, the following software was utilized for statistical analyses: IBM SPSS version 27.0, R Statistics version 4.3.1 (R Foundation for Statistical Computing), and GraphPad Prism version 8.0.0. All hypothesis tests were two‐sided. *p*‐value < 0.05 was considered statistically significant.

## AUTHOR CONTRIBUTIONS

Z.T., X.W., and Y.S. contributed to the study conception and design. Blood sample collection was conducted by Z.S. and W.J. Data collection were performed by Z.S., W.C., and K.S. Data analysis were conducted by Z.L. The first draft of the manuscript was written by Z.L., Z.S., and W.J., and all authors commented on the manuscript. All authors read and approved the final manuscript for submission.

## CONFLICT OF INTEREST STATEMENT

The authors declare no conflicts of interest.

## ETHICS STATEMENT

This study was approved by the Ethics Committee of Zhongshan Hospital, Fudan University (IRB number: B2018‐264). All participants enrolled in this study signed the informed consent according to the requirements of the Ethics Committee of Zhongshan Hospital, Fudan University.

## Supporting information



Supporting Information

## Data Availability

The raw data for this study are available in a publicly accessible database: CNCB‐NGDC (code: HRA013699). It should be noted that DNA sequencing results contain sensitive personal data and thus need to be analyzed and utilized in accordance with data protection policies.
