# Supplementary material for: Circulating tumor DNA analysis for prediction of prognosis and molecular insights in patients with resectable gastric cancer: results from a prospective study
Source: MedComm (2020). 2025 Jan 19;6(2):e70065. doi: 10.1002/mco2.70065 (PMC11742430; doi:10.1002/mco2.70065)
Supplement: Supplementary file 1 — Supporting Information [file MCO2-6-e70065-s001.docx]

**Circulating Tumor DNA Analysis for Prediction of Prognosis and Molecular Insights in Patients with Resectable Gastric Cancer: Results from a Prospective Study**

Zheng Liu^1,2,4,#^, Zhongyi Shi^1,2,#^, Wenchao Jiang^1,2,#^, Zhenbin Shen^1,2^, Weidong Chen^1,2^, Kuntang Shen^1,2^, Yihong Sun^1,2,3^, Zhaoqing Tang^1,2,3,*^, Xuefei Wang^1,2,3,*^

1. Department of Gastrointestinal Surgery, Zhongshan Hospital, Fudan University, Shanghai, China

2. Gastric Cancer Center, Zhongshan Hospital, Fudan University, Shanghai, China

3. Department of General Surgery, Zhongshan Hospital (Xiamen branch), Fudan University, Shanghai, China

4. Shanghai Medical College, Fudan University, Shanghai China

# Zheng Liu, Zhongyi Shi and Wenchao Jiang contributed equally to this work.

***Correspondence to:** Zhaoqing Tang, Department of Gastrointestinal Surgery, Zhongshan Hospital, Fudan University, Shanghai 200032, China, Email: tang.zhaoqing@zs-hospital.sh.cn; Xuefei Wang, Department of Gastrointestinal Surgery, Zhongshan Hospital, Fudan University, Shanghai 200032, China, Email: wang.xuefei@zs-hospital.sh.cn.


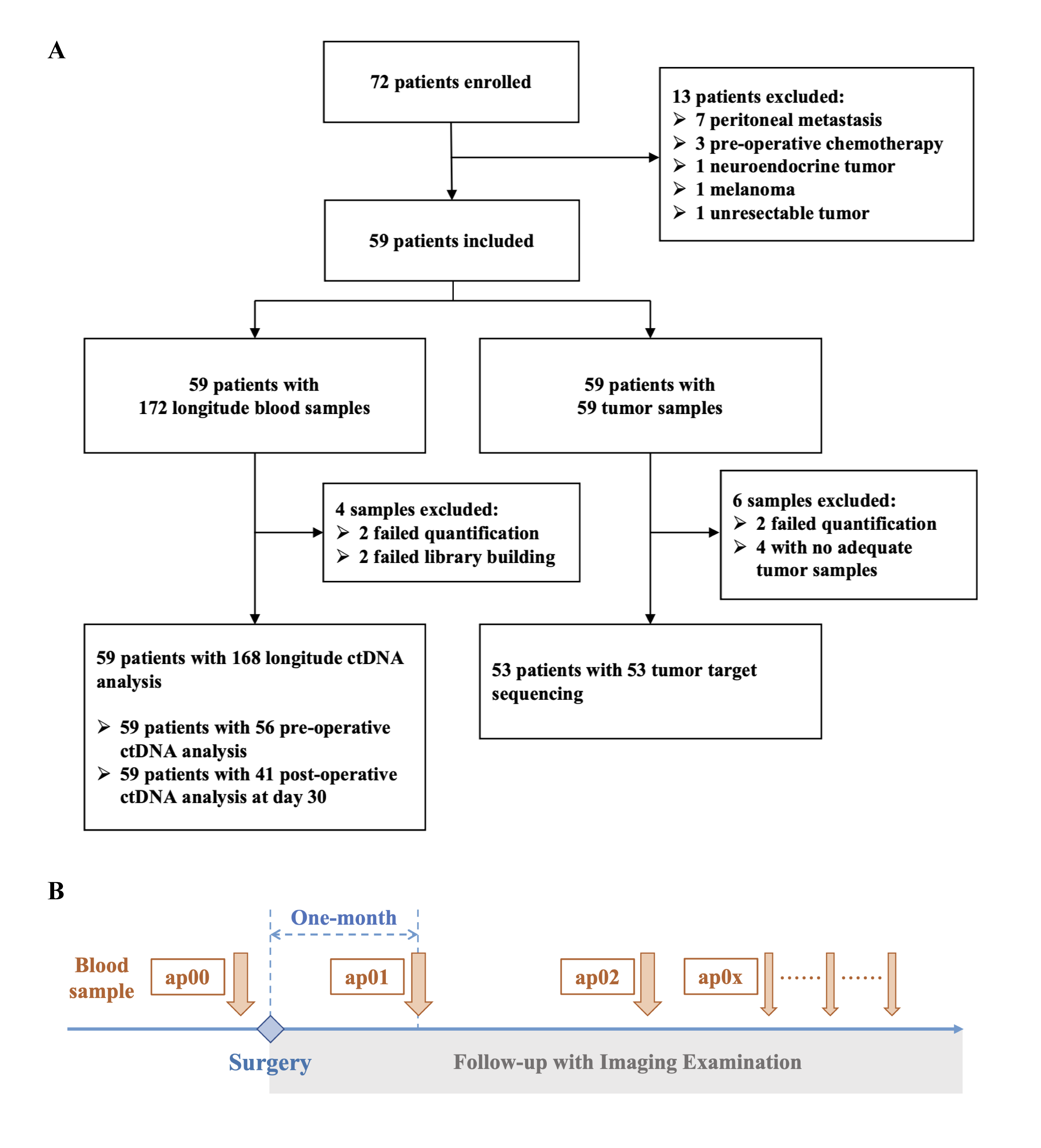


**Figure S1. Study design**

Flowchart of patient enrollment (**A**). Schematic diagram of sample collection in peri-operative period and follow-up (**B**).

Abbreviations: ctDNA, circulating tumor DNA. Ap00: Pre-operative (baseline). Ap01: one-month post-operative.


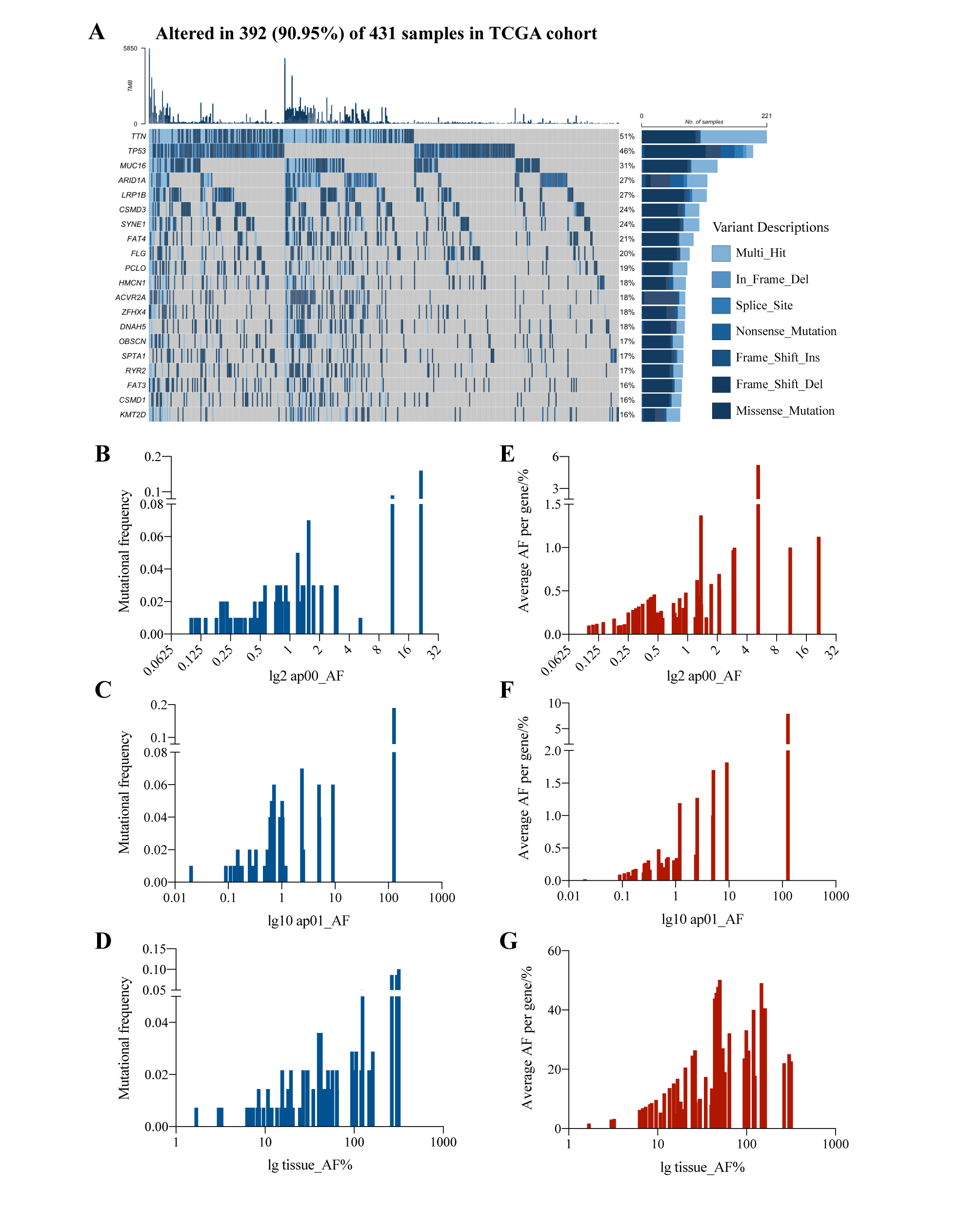


**Figure S2, related to Figure 1. The mutational landscape of genomic alterations detected in ctDNA of plasma samples and tumor tissue.**

Waterfall plot exhibited single nucleotide variation (SNV) in 431 tissue samples in TCGA stomach adenocarcinoma (STAD) cohort (**A**). The mutational frequency approximately increased in direct proportion to ctDNA AF in ap00 (**B**), ap01 (**C**) and in tumor tissue (**D**). The average AF per gene approximately increased in direct proportion to ctDNA AF in ap00 (**E**), ap01 (**F**) and in tumor tissue (**G**).

Abbreviations: TMB, tumor mutation burden.

**
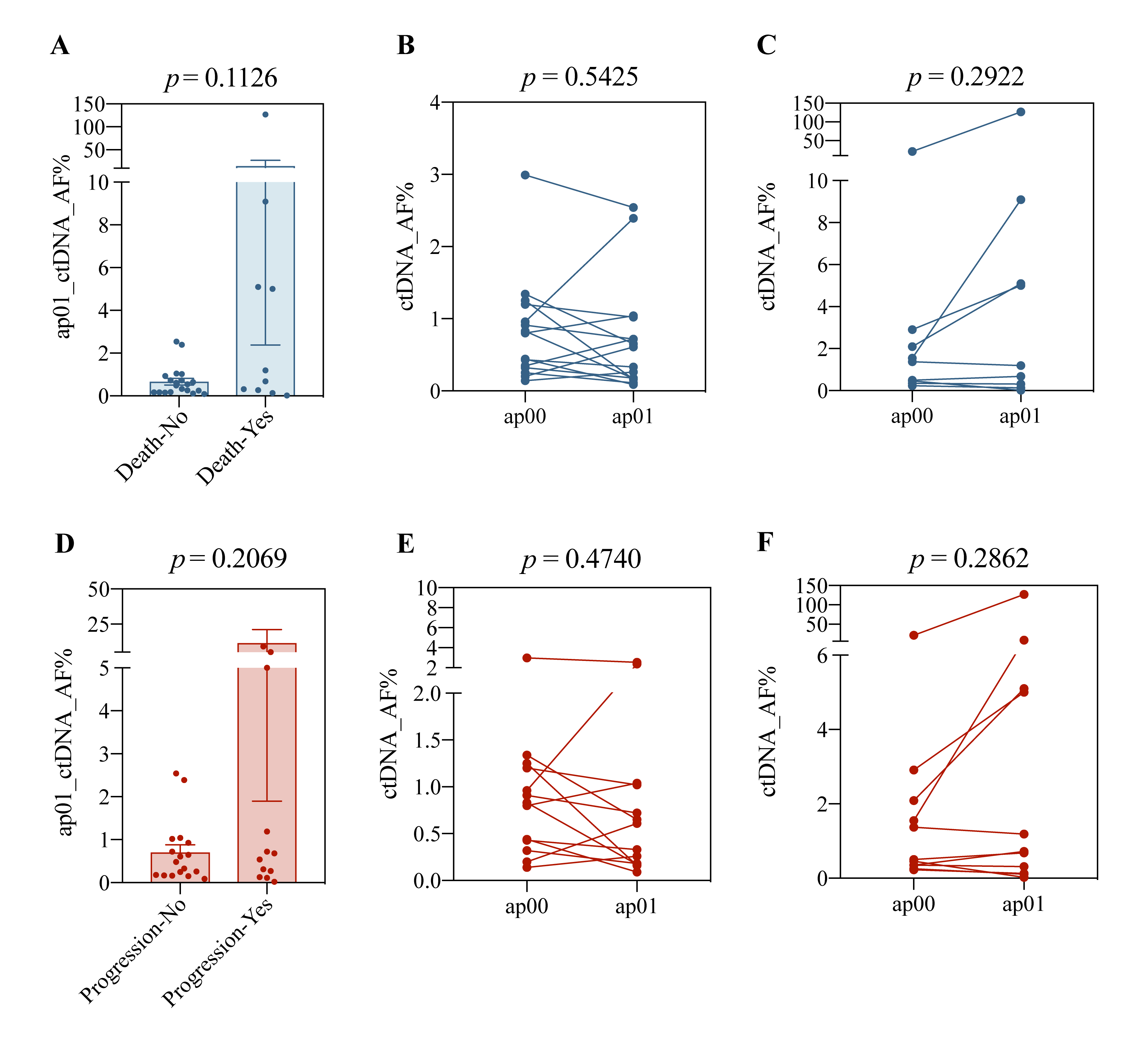
**

**Figure S3, related to Figure 2. The correlations between survival status and levels of ctDNA AF in post-operative plasma samples in patients with resectable gastric cancer.**

There was no significant difference in ap01 ctDNA allele fraction (AF) level between patients who died (Death-Yes) and who survived (Death-No) during 3-year follow-up (**A**). The level of ctDNA AF in plasma samples showed no significant change before surgery (ap00) and one month after surgery (ap01), whether in patients who survived (**B**) or died (**C**) during 3-year follow-up. The best cut-off value of ap01 ctDNA AF was 2.54%. The p value of Log-rank test was indicated. There was no significant difference in ap01 ctDNA AF level between patients who had progression disease (Progression-Yes) and who achieved stable disease (Progression-No) during 3-year follow-up (**D**). The level of ctDNA AF in plasma samples showed no significant change before surgery (ap00) and one month after surgery (ap01), whether in patients who did not develop progression(**E**) or had progression (**F**) during 3-year follow-up.


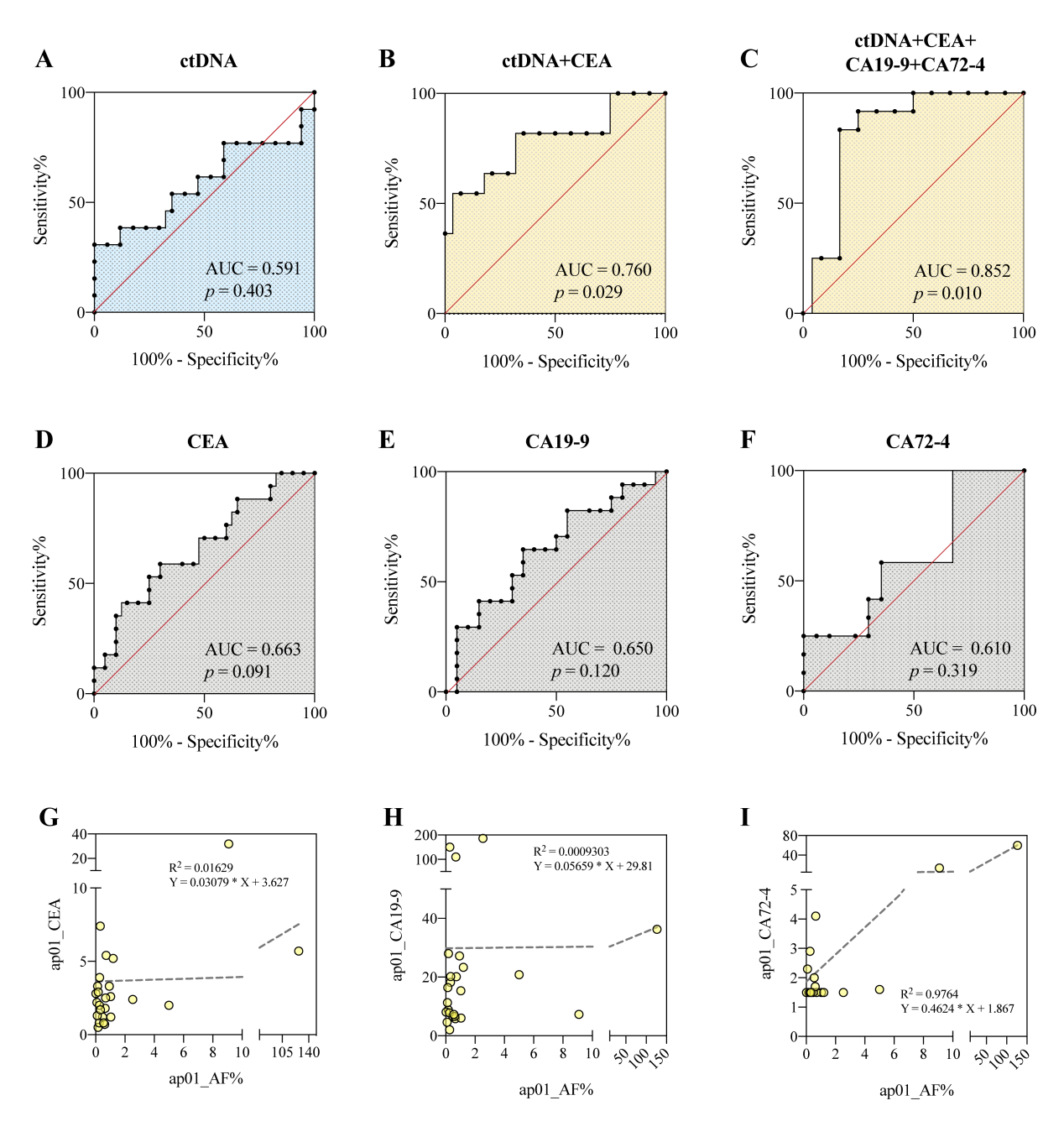


**Figure S4, related to Figure 3. One-month post-operative plasma ctDNA level as a prospective factor for predicting recurrence.**

ROC analysis of net ctDNA (**A**), the combination of ctDNA and CEA (**B**), the combination of ctDNA and the other four conventional tumor markers for gastric cancer (**C**), CEA (**D**), CA19-9 (**E**), and CA72-4 (**F**) for 3-year recurrence prediction with AUC and p value presented. There was no statistically significant correlation between ctDNA AF and CEA (**G**)/CA19-9 (**H**) levels at ap01. At ap01, a high and positive correlation of ctDNA AF with CA72-4 (**I**) was determined, with R^2^=0.9764 in the regression analyses.


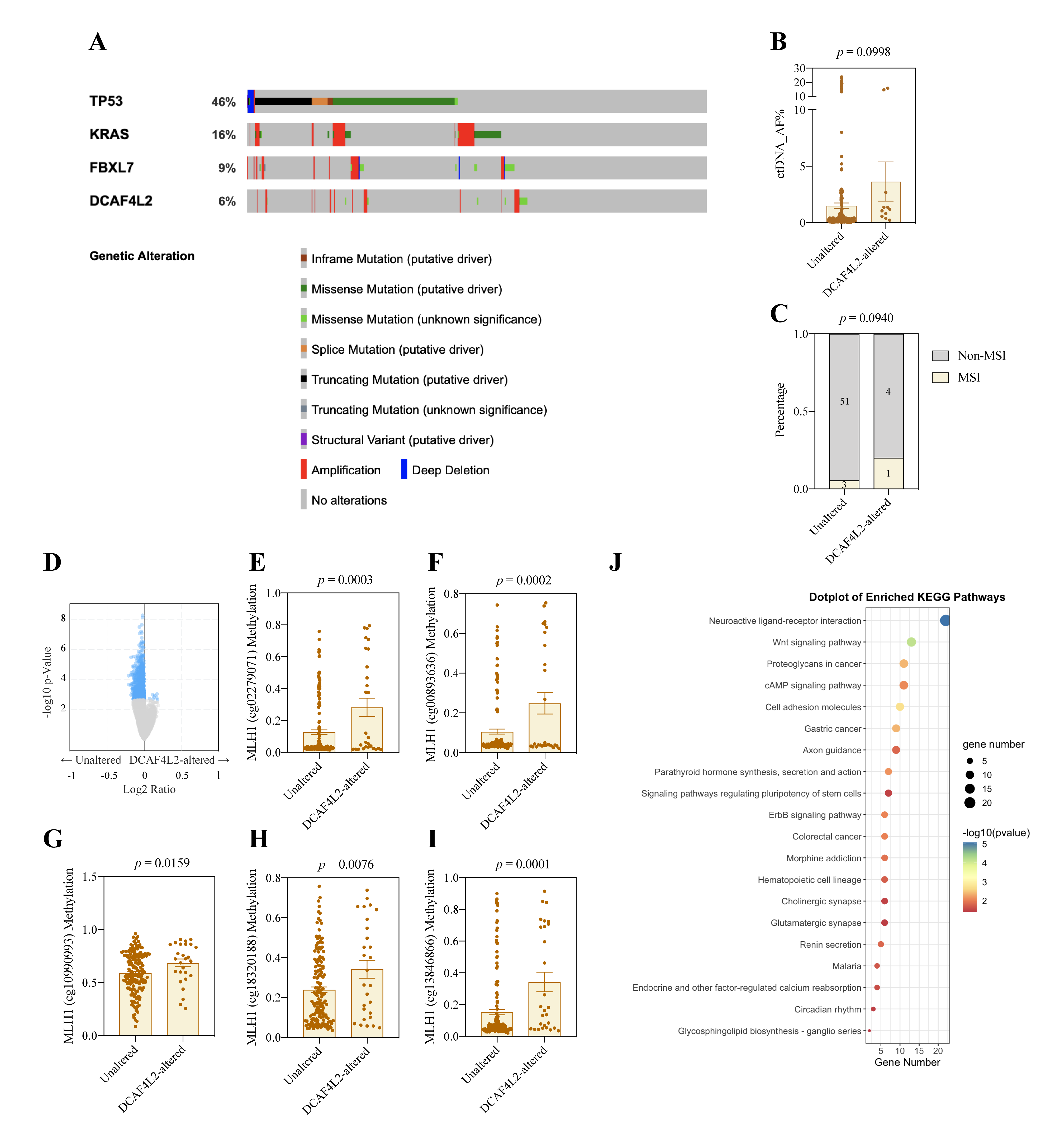


**Figure S5, related to Figure 5. *DCAF4L2* was identified as a crucial gene for poor prognosis in patients with gastric cancer.**

The mutation frequency and genetic alteration type of 4 candidate genes in 1213 tissue samples of gastric cancer from TCGA STAD cohort (including 3 studies: Nature, 2017; PanCancer Atlas; Firehose Legacy; Nature 2014) were presented (**A**). Patients in *DCAF4L2*-altered group (n=184) tended to have higher ctDNA AF level than ones in unaltered group (n=11) in our cohort (**B**). There was a tendency that patients in DCAF4L2-altered group were likely to have microsatellite instability (MSI) type of gastric cancer (**C**). The number labeled on the bar represented the number of samples included. Total methylation level in *DCAF4L2*-altered and unaltered group was displayed as a volcano plot (**D**). Patients in *DCAF4L2*-altered group (n=158) had significantly higher methylation level of MLH1 via several probes than unaltered group (n=28) (**E-I**). Enriched Kyoto Encyclopedia of Genes and Genomes (KEGG) pathways of the differentially methylated genes in DCAF4L2-altered and unaltered group were depicted (**J**).

**Table S1. Summary of predictive efficacy for ap01 conventional tumor markers and other associated clinicopathologic characteristics.**

| **ap01** | **CEA+CA19-9+CA72-4** | **ctDNA+CEA+CA19-9**  **+CA72-4+pTNM** | **ctDNA+CEA+CA19-9**  **+CA72-4+Tumor size** |
| --- | --- | --- | --- |
| **AUC** | 0.779 | 0.952 | 0.952 |
| **Std. Error** | 0.091 | 0.046 | 0.046 |
| **95%CI** | 0.600 - 0.958 | 0.863 – 1.042 | 0.863 – 1.042 |
| ***p*-value** | 0.015 | 0.001 | 0.001 |
| **Cut-off** | 0.265 | 0.205 | 0.146 |
| **Sensitivity** | 0.700 | 1.000 | 1.000 |
| **Specificity** | 0.789 | 0.833 | 0.833 |

**Abbreviations**: AUC, area under curve; Std. Error, standard error; CI, confidence interval; pTNM, pathological Tumor-Node-Metastasis
